# A 3-piece penile prosthesis salvage in the presence of late-onset infected hematoma: Clinical, radiological and intraoperative findings—A case report

**DOI:** 10.1016/j.ijscr.2019.10.084

**Published:** 2019-11-06

**Authors:** Moayid Fallatah, Manerh Bin Mosa, Ahmed Aljuhayman, Naif Alhathal

**Affiliations:** a Department of Urology, King Faisal Specialist Hospital and Research Center, Riyadh, Saudi Arabia; b Division of Urology, King Abdulaziz Medical City, Ministry of National Guard – Health Affairs, Riyadh, Saudi Arabia

**Keywords:** Penile implant, Complications, Infection, Hematoma, Salvage, Case report

## Abstract

**Introduction:**

Erectile dysfunction (ED) is a common health problem affecting 30% of young men worldwide. Despite the availability of non-invasive lines of management, penile prosthesis insertion is considered as a definite solution for ED. Despite strict perioperative measures, infection still complicates around 3% of penile prosthesis surgeries.

**Presentation of case:**

This is a case of a 36-year-old male who had an infected scrotal hematoma that led to prosthesis malfunction due to the inability to locate the pump to activate and deactivate the device. Intraoperatively, the prosthesis was salvaged after hematoma evacuation and ensuring a good device functionality.

**Discussion and conclusion:**

Infected penile prostheses are usually either removed completely with a new device insertion a few months later or exchanged at the same setting with vigorous wound washout. We suggest salvaging penile implants which are surrounded by infected hematomas in selected patients who don’t manifest systemic signs of infection. This approach will help in cost reduction and avoiding further intraoperative complications.

## Highlights

• Penile prosthesis infection is a serious complication often managed radically.
• In selected patients, infected prosthesis can be salvaged without removal.
• Wound washout with antimicrobials and antiseptics is a key component of the management.

## 1 Introduction

Erectile dysfunction (ED) is a common health problem affecting 30% of young men worldwide [1]. Despite the availability of various lines of medical management, penile prosthesis insertion is considered a definite solution for ED. Historically, penile implants were first introduced in 1964 by Lash et al. for the management of Peyronie’s disease [2]. Regarding ED, penile prostheses have been used as an option since the mid-seventies [3]. Regardless of strict prophylactic and preventive measures, infection is still considered the most frequent and serious sequela that complicates 3% of penile prosthesis surgeries [4]. When it happens, that necessitates the removal of the entire inflatable penile prosthesis (IPP) followed by re-implantation 3–6 months later, or removal of the IPP, wound washout and implanting a new prosthesis immediately (salvage technique) [5]. There are a few papers in the literature which reported successful conservative management without removal of the infected prosthesis [[6], [7], [8]]. We report a case of a malfunctioning penile prosthesis with an infected scrotal hematoma surrounding the pump. The patient was managed at an academic hospital successfully by hematoma evacuation, intraoperative washout and postoperative antibiotics without removing the prosthesis. This case is reported in line with the SCARE criteria [9].

## 2 Presentation of case

**Fig. 1:**
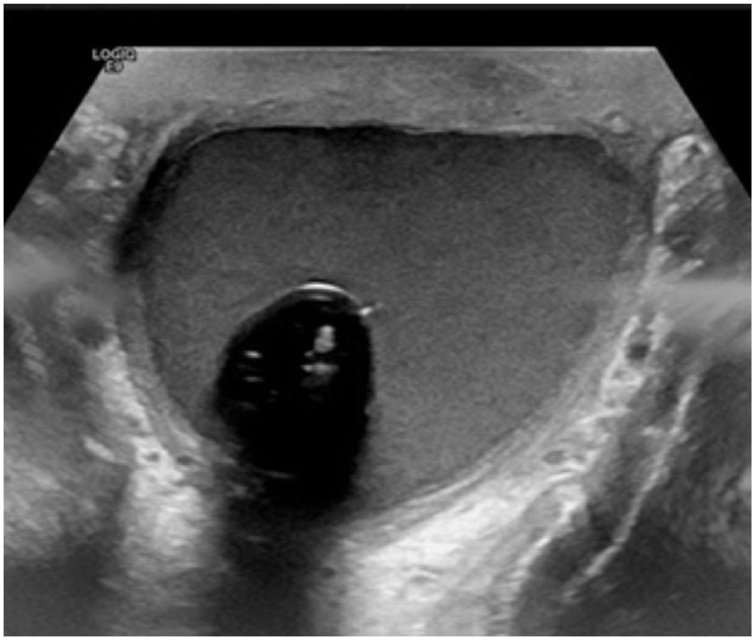
Scrotal ultrasound showing a scrotal collection 1 month after the primary procedure.

**Fig. 2:**
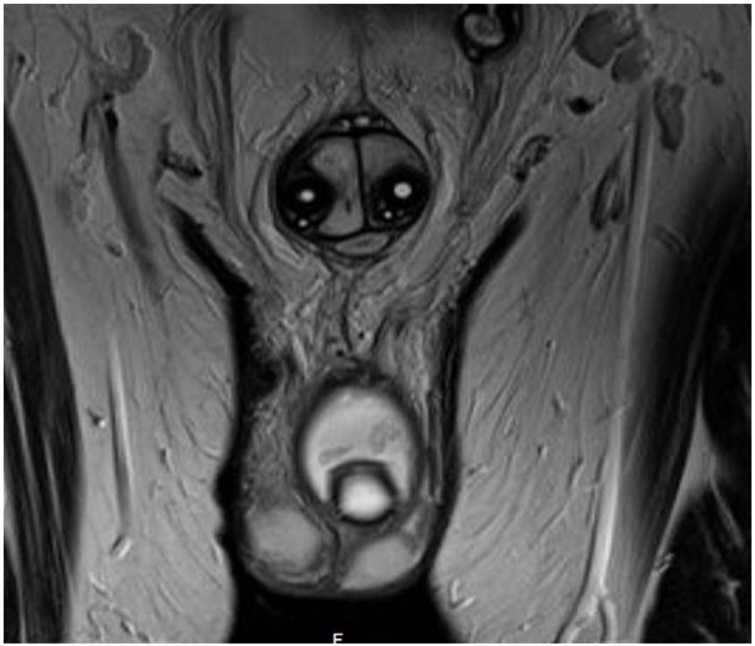
Pelvic MRI showing a scrotal collection around the pump.

**Fig. 3:**
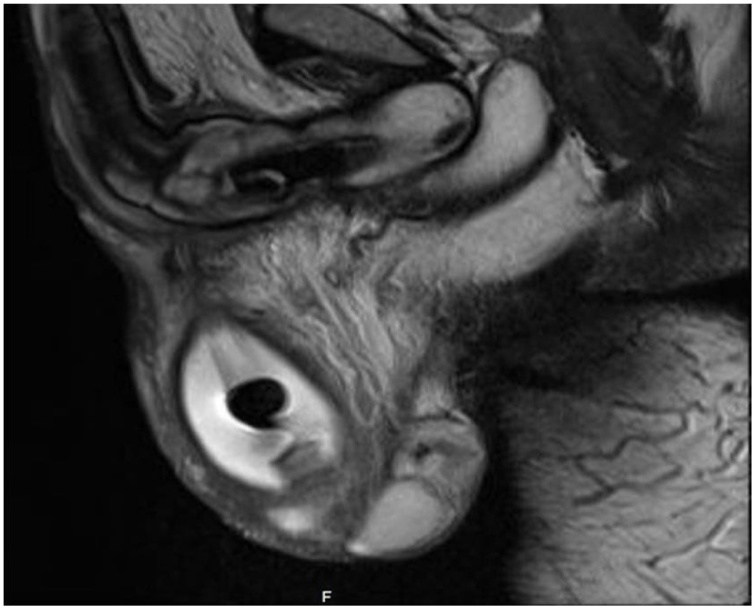
Pelvic MRI showing a scrotal collection around the pump.

We present a 36-year-old man, smoker with no known chronic medical illnesses, who had a history of idiopathic priapism for which he underwent a distal (Al-Ghorab) shunt surgery 3 years ago. Consequently, the patient presented to our clinic complaining of ED. As a result, he was started on Tadalafil and Alprostadil intracorporeal injections for 3 years but without satisfying erections. Thus, the choice of inserting a 3-piece penile prosthesis was discussed with the patient and he agreed. In June 2018, the patient underwent penile prosthesis insertion (Coloplast Titan®). Intraoperatively, severe corporal fibrosis was noted and dilated successfully. The procedure went uneventfully without complications. The patient was admitted for pain control and a 4-day course of intravenous Vancomycin and Gentamycin. During the hospital stay, no scrotal swelling or tenderness were noted, and the patient was discharged on Ciprofloxacin plus Cephalexin for 14 days. A month later, the patient presented with a painless scrotal swelling. However, no signs of systematic infection were detected. Local examination revealed an intact surgical scar with no overlying skin changes or discharge. Additionally, a small left scrotal confined collection was felt. Scrotal ultrasonography revealed 5.6 × 3.3 × 3.9 cm scrotal hematoma surrounding the prosthesis pump (Fig. 1). As there were no signs of infection and the device was functioning properly, the patient was managed conservatively with scrotal support and he was instructed not to use the device until the resolution of the hematoma. On the following visit, hematoma decreased in size, prosthesis recycling was performed successfully, and the patient was allowed to resume sexual intercourse. Two months later, the patient presented with a painless large scrotal swelling with the inability to use the pump due to the surrounding collection. On examination, there were no signs of local or systemic infection. A large, painless and firm scrotal swelling at the site of the pump was palpated. A pelvic MRI showed a 6.4 × 3.6 × 3.9 cm scrotal collection with findings suggestive of an underlying infectious process (Fig. 2, Fig. 3). The choice of elective scrotal exploration with a possible exchange of the implant was discussed with the patient, and he agreed after all risks and benefits were explained.

**Fig. 4:**
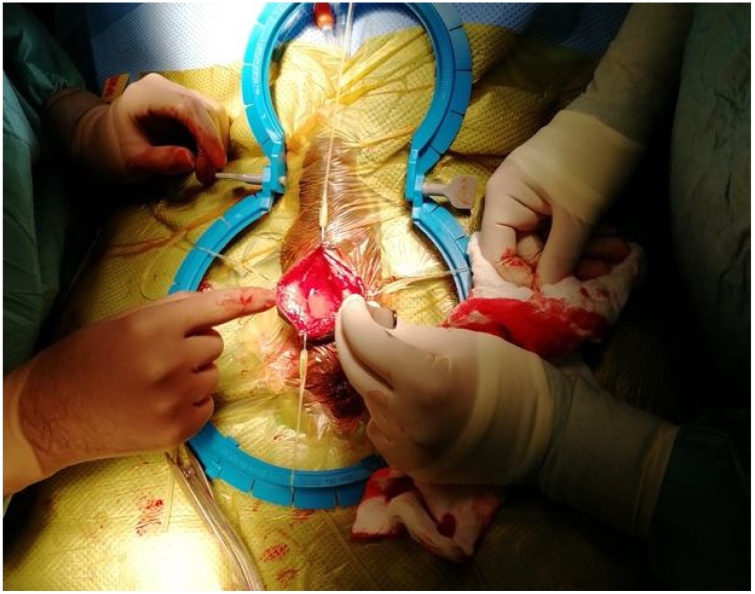
Purulent discharge noted upon opening the pump space.

**Fig. 5:**
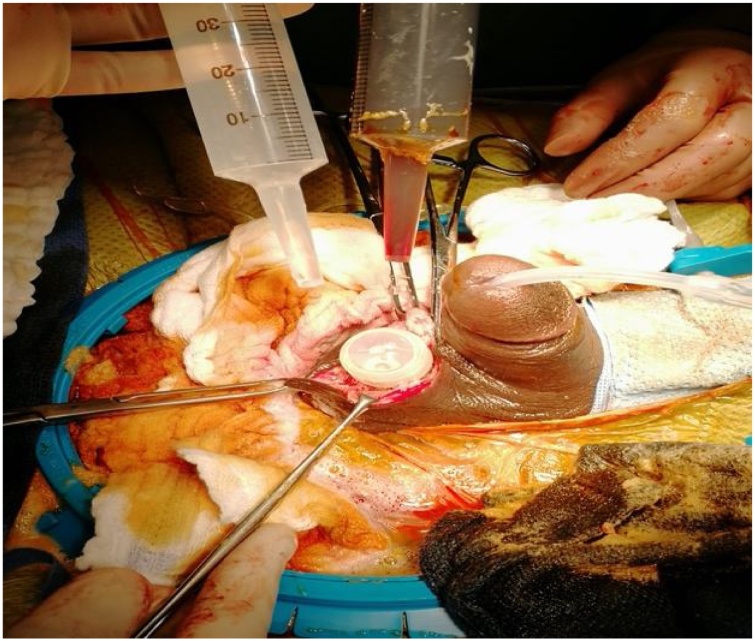
Antimicrobial and antiseptic solutions used in irrigation and the penis is fully erected after a successful prosthesis recycling.

Under general anesthesia, a penoscrotal incision was made. The pump was covered by a fibrous layer which was incised sharply. Upon opening the capsule surrounding the pump, blood poured out and a sample was taken for culture (Fig. 4). Complete hematoma evacuation and excision of the cavity was done, and the device was recycled successfully multiple times with no evidence of malfunction (Fig. 5). Accordingly, the decision was made not to replace the device. Vigorous scrotal washout was done using 1-liter of antimicrobial solution (Vancomycin and Gentamycin), povidone and hydrogen peroxide. Then, a new space was created for the pump in the right scrotal side. Eventually, size 10 French Jackson-Pratt drain was inserted, and the scrotal layers were closed using vicryl suture.

The intraoperative culture came back positive for Staphylococcus Lugdunensis, so the patient was shifted from intravenous Vancomycin and Gentamycin to oral Clindamycin 600 mg three times a day for 3 weeks. The patient was discharged on day 7 postoperatively in a good condition. The patient is being followed up postoperatively for 2 months now with no evidence of hematoma recurrence or prosthesis malfunction.

## 3 Discussion

Penile prostheses are considered a definite line of management for refractory ED [10]. In uncomplicated surgeries, penile prostheses are associated with a high level of satisfaction (>85%) among patients and their partners [11]. Similar to any prosthetic surgery, complications can arise at the time of the placement of the prosthesis or at a later date. In a penile prosthesis surgery, complications usually are related to infection and\or mechanical failure, and when it happens, it is associated with high costs and patient dissatisfaction [7]. Moreover, the cost of treating an infected IPP has been estimated to be more than 6 times the cost of the original implant [12].

It is well known that systemic antibiotics have limited penetration to the infected prosthesis due to bacterial biofilms [13]. Therefore, surgical interventions consisting of removing all device components as well as any permanent sutures or graft material used during corporeal reconstruction are the gold standard of care in symptomatic penile prosthesis infections. Classically, infected implants were managed by removing the prosthesis with antimicrobial washout of the spaces and re-insertion of implants a few months later, however, this approach was associated with great morbidity caused by the extensive corporal fibrosis leading to difficult re-implantation [14]. The salvage approach where a new device is re-implanted in the same setting of the removal was first described by Brantley Scott’s group. Their attempt of immediate re-insertion of the prosthesis was successful in 36 out of 44 patients (83.7%) [15]. Similar success rate (82%) was reported by Mulcahy in the early beginning of this century [5].

As replacing the prosthesis results in high financial cost and expose the patient to more surgical complications, some surgeons suggested salvaging the IPP if the infection is limited to the local tissue. Deroue et al. were the first to demonstrate the success of conservative management while retaining the infected implant in 3 patients who were managed by local and systemic Clindamycin. The wounds healed in 2–3 weeks, and the implants were working properly afterward [6]. Recently, Luján et al. reported another two cases of diabetic patients who presented with scrotal wound small separation with clear discharge [7]. Their cultures revealed a Staphylococcus Aureus and Staphylococcus Epidermidis. After 2 weeks of unsuccessful oral antibiotics course, both patients had their implants successfully salvaged by surgical debridement of all devitalized tissue around the pump and by using high pressure pulsated lavage (Interpulse; Stryker Corp, Kalamazoo, MI, USA). This treatment modality has been successfully used in orthopedic implant infection to achieve optimal necrotic tissue and biofilm removal with a success rate of 86.5% [16]. Vancomycin, hydrogen peroxide and povidone-iodine were used for irrigation. Eventually, multilayered surgical closure was performed, and a drainage tube was kept for 24 h. Both patients were discharged 24 h postoperatively on a 10-day course of oral antibiotics. Total resolution of the symptoms was achieved, and the implants worked properly after 20 and 36 months.

With regards to our case, we opted not to remove or replace the prosthesis as there was no intraoperative evidence of mechanical failure and there was no connection between the hematoma and the prosthesis. Additionally, the patient was totally asymptomatic with no signs of local or systemic infection. Intraoperatively, irrigation was done using 1-liter of antimicrobial solution (Vancomycin and Gentamycin). Multilayered closure was done after ensuring a good prosthesis functionality. Jackson-Pratt drain was placed to ensure good drainage and it was removed after 2 days. Our patient remained in the hospital to receive intravenous antibiotics (Vancomycin and Gentamycin) waiting for the final culture and sensitivity result which came positive for Staphylococcus Lugdunensis. The patient was discharged on oral Clindamycin for 21 days as per infectious disease recommendation. The patient is being followed up postoperatively for 2 months with no evidence of hematoma recurrence or prosthesis malfunction.

## 4 Conclusion

While there are only a few reported cases with successful penile prosthesis salvage, it is reasonable to consider salvaging prostheses which are surrounded by late-onset infected hematoma in patients without systemic infection manifestations. That will help in cost reduction and avoiding further complications that may be caused by the new device insertion.

## Sources of funding

This research did not receive any specific grant from funding agencies in the public, commercial, or not-for-profit sectors.

## Ethical approval

Case reports are exempted from IRB/ethical approval at the institution.

## Consent

A written consent to publish the case with operative and radiological images was obtained from the patient.

No identifying details are included in the manuscript, and the patient images don’t contain identifying features.

## Author contribution

Naif Alhathal: Writing - review & editing, Investigation, Supervision.

Moayid Fallatah: Writing - review & editing, Investigation.

Ahmed Aljuhayman: Writing - review & editing, Investigation.

Manerh Bin Mosa: Writing - original draft, review & editing, Investigation.

## Registration of research studies

Case reports are exempted from registration.

## Guarantor

Dr. Naif Alhathal.

## Provenance and peer review

Not commissioned, externally peer-reviewed

## Declaration of Competing Interest

All authors declare no conflict of interests.
